# A trend prevalence of visceral Leishmaniasis in West Armachiho District, Amhara Region, Northwest Ethiopia

**DOI:** 10.1186/s40794-020-00125-z

**Published:** 2020-11-27

**Authors:** Addisu Gize, Addisu Workineh, Taddesse Hailu

**Affiliations:** 1 Department of Microbiology, St. Paul’s Hospital Millennium Medical College, Addis Ababa, Ethiopia; 2 Health Promotion and Disease Prevention Directorate, Amhara National Regional State Health Bureau, Bahir Dar, Ethiopia; 3 Department of Medical Laboratory Science, College of Medicine and Health Sciences, Bahir Dar University, Bahir Dar, Ethiopia

**Keywords:** Visceral Leishmaniasis, Trend, Prevalence, Armachiho

## Abstract

**Background:**

Visceral leishmaniasis (VL) is a disease caused by an obligate intracellular protozoan parasite that affects animals and humans. An estimated 3.2 million people are at risk of VL, and 3700–7400 cases occur annually in Ethiopia. The highest numbers of VL cases have been previously reported from the North Western parts of the country, especially in West Armachiho District. The aim of this study was to determine the trend prevalence of VL at the study area.

**Methods:**

Health center based retrospective data were collected to determine the trend prevalence of VL among patients who had blood examination from January 2010 to August 2015. The blood samples were collected by finger pricking and the infections were confirmed by using rK_39_ antibody test.

**Result:**

Of the 9299 VL suspected cases, 1948 (21%) were positive for rK_39_ antibody test. Of these, 1757 (90.2%) were primary kala-azar cases, 167 (8.6%) were relapse and the remaining 24 (1.2%) were post kala-azar dermal leishmaniasis cases.

**Conclusions:**

The prevalence of VL is still high in the study area. Therefore, early case detection, diagnosis, treatment, and timely analysis are essential.

## Introduction

Leishmaniasis is a disease caused by a parasitic protozoan infection of genus *Leishmania*. The disease is endemic in 98 countries [1], as 200,000–400,000 new cases and 20,000–30,000 deaths occur annually. Leishmaniasis is most represented in East Africa [2], and most prevalent in the Northern lowlands, and Southern parts of Ethiopia [3]. In Ethiopia, an estimated 3.2 million people are at risk of acquiring VL, and 3700–7400 cases occur annually [4, 5]. The highest number of VL cases has been previously reported in the north western Ethiopia including West Armachiho District [4, 6].

The prevalence of VL is dynamic as its mode of transmission changes according to the environment, socio-economic status, and immune status of the population [7].

Marked increase in VL cases are associated with migration of non-immune labourers, and Human Immunodeficiency Virus (HIV)-VL co-infection, in the north western and lowland parts of the country respectively [8, 9].

The clinical manifestation of VL patients includes: fever (more than 2 weeks), fatigues, weakness, loss of appetite, weight loss, enlarged lymph nodes, hepatosplinomegally and sometimes bleeding [10]. Post Kala-azar Dermal Leishmaniasis (PKDL) is a consequence of VL and infected cases are considered to be a potential source of kala-azar infection [11].

The prevalence of PKDL in Ethiopia is higher in people co-infected with HIV and VL [12].

*Leishmania donovani* infected cases should be properly identified from other febrile cases, and with highly sensitive and specific diagnostic laboratory tests. Demonstration of *Leishmania* amastigotes microscopically in tissue aspirates is a confirmatory diagnostic technique, however its application is limited in the study area due to several factors, like personal bias among laboratory professionals [13]. As a result, there is a definite need for continued use of rK_39_ test kits for the primary diagnosis of VL.

Using the recombinant product of K_39_ (rK_39_) antigen to test the corresponding antibody is a very crucial diagnostic approach in implementing cost effective treatment and sustainable use of antileishmanial drugs. It is very important in peripheral health care systems of the country since it requires minimal trained personnel and resource investments. Timely analysis of the trends of VL using rK_39_ in remote areas and resource poor countries like Ethiopia alleviates the disease burden of the community. Therefore, the aim of this retrospective study was to determine a 5 year and 6 month trend analysis of VL prevalence at Abdurafi health center, northwest Ethiopia.

## Methods

### Study area and population

The study was conducted in Abdurafi Health Center located in West Armachiho district, Amhara region, North Gondar, Ethiopia. The area is 950 km from the capital city of Addis Ababa.

West Armachiho District is one of the VL endemic districts which covers 3335.29 square kms and is administratively divided into 10 rural and four urban sub-districts (kebeles). The area has an elevation which ranges between 550 and 1600 m above sea level. Based on the 2007 Census conducted by the Central Statistical Agency of Ethiopia, the district has a total population of 45,257 of which 22,173 are females [14]. The district has 14 government health facilities (one rural hospital, 10 health posts, and three health centers, including Abdurafi health center), and 24 clinics (23 private and 1 non-government organization (NGO)).

The health center is one of the largest leishmaniasis treatment centers in northwest Ethiopia, supported by the NGO, Medicins sans Frontières. Diagnosis of VL in the health center was performed according to the WHO guidelines. A health center based retrospective study was conducted on VL suspected cases who attended the Abdurafi health center from January 2010 to June 2015. The target population was 9299 VL suspected cases.

### Clinical and laboratory diagnosis

The rK39 antibody test kit uses a highly conserved kinesin-related protein-encoding gene, which contains a repetitive 117-bp sequence that encodes for 39 amino acid residues (K39), and is conserved in all of the VL-causing isolates [15]. The recombinant product of K_39_ (rK_39_) has proven to be a very sensitive and specific antigen used for the serodiagnosis of VL in different endemic foci. The test is simple, rapid (10 min), relatively inexpensive, requires no other reagents or instruments and can be performed in the field by the paramedics [16]. In Ethiopia, detection of *L. donovani* parasites in blood was conducted according to a standard operating procedure (SOP) in each health institution throughout VL endemic areas. Therefore, in this study we collected the VL confirmation test data for 5 years and 6 months (January 2010 to June 2015) retrospectively from Abdurafi health center.

### Statistical methods

Data were entered into excel and transported to SPSS. Analysis was performed by SPSS version 20 statistical software package. Frequency and percentage were calculated for the study variable. Chi-square, *p*-value and two tail Fisher‘s exact test was used to calculate and determine significance levels. In all statistical tests, the differences were considered to be statistically significant if p-value was less than 0.05.

### Ethical consideration

The ethical review committee of Amhara National Regional State Health Bureau and College of medicine and Health Sciences, Bahir-Dar University approved the project. The researchers obtained informed consent from the head of the Abdurafi health center.

## Results

### Socio-demographic information

Over the last 5 years and 6 months, 9299 VL suspected cases were examined at the Abdurafi Health Center, of which 1948 (21%) were positively confirmed. Of the total VL confirmed cases, 1757 (90.2%) were reported primary Kala-azar (PKA) cases. Most of the suspected cases were above 15 years of age, and monthly number of cases ranged from 3 to 71 with an average of 30. A large population was at risk for acquiring PKA in 2015, as it is described in Table 1.

**Table 1:** VL attack rate per 1000 populations of Abdurafi health center, West Armachiho District, Amhara, Ethiopia, 2010–2015

Year	Attack rate per 1000 population												Population at risk
	Jan	Feb	Mar	Apr	May	June	July	Aug	Sep	Oct	Nov	Dec	
2010	0.76	0.57	0.82	0.57	0.33	0.57	1.17	0.95	1.23	1.12	1.06	1.58	36,733
2011	0.73	0.83	0.78	0.44	0.52	0.52	0.42	0.52	0.55	0.62	0.88	1.14	38,438
2012	0.60	0.62	0.82	0.75	0.65	0.50	0.40	0.57	0.55	0.42	1.25	0.92	40,143
2013	1.03	0.50	0.67	0.29	0.41	0.48	0.31	0.50	0.36	1.08	0.55	0.57	41,825
2014	1.44	1.00	1.62	0.96	1.35	0.68	0.59	0.48	0.71	1.07	0.78	0.66	43,825
2015	0.77	0.82	0.66	0.07	0.66	0.24							45,257

**Fig. 1:**
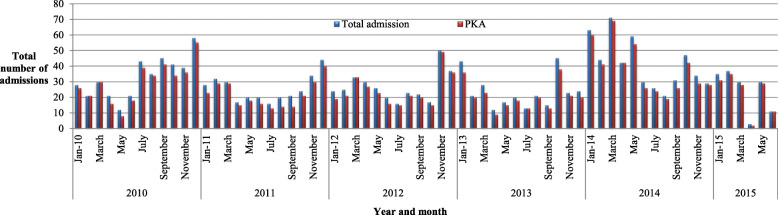
Total number of admissions and primary cases in Abdurafi health center, West Armachiho District, Ethiopia, 2010–2015

**Fig. 2:**
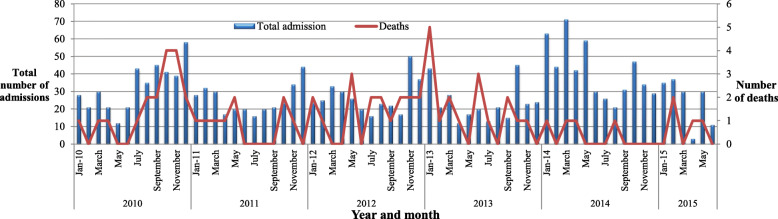
Total number of admissions and deaths in Abdurafi health center, West Armachiho District, Ethiopia, 2010–2015

**Fig. 3:**
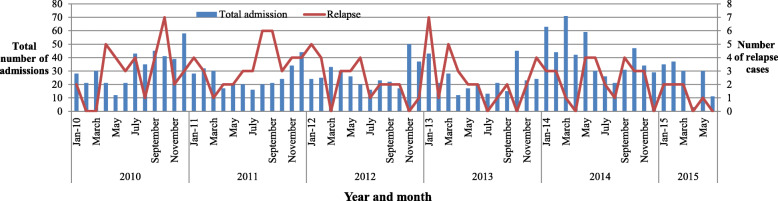
Total number of admissions and relapse cases in Abdurafi health center, West Armachiho District, Ethiopia, 2010–2015

**Fig. 4:**
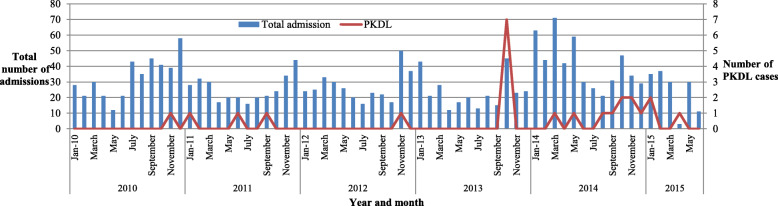
Total number of admissions and post kala-azar dermal leishmaniasis cases in Abdurafi health center, West Armachiho District, Ethiopia, 2010–2015

There was a higher number of VL admission cases in 2014 (484, 24.9%) as compared to 2010 (403, 20.7%) (Fig. 1). Additionally, the highest death rate was observed in 2013 (17, 5.8%) (Fig. 2). Among the admitted cases, 167 (8.6%) cases were reported as relapse (Fig. 3) and the remaining 24 (1.2%) were Post Kala-azar Dermal Leishmaniasis (PKDL) cases. The highest number of admitted cases who developed PKDL was 7 (2.4%) in the year 2013 (Fig. 4).

## Discussion

VL is a major health problem especially in developing countries like Ethiopia [17].

In the present study, the overall prevalence of VL was 1948 (21%). The majority, 1695 (87%), of VL confirmed cases were above 15 years of age. This could be due to increased exposure to sand flies during daily activities, such as tending domestic animals outdoors [18, 19]. The highest convenience sample prevalence of VL cases was reported in 2014 at 484 (24.9%) and 2010 at 403 (20.7%).

In the previous survey, environmental factors, host factors, and migration of non-immune labourers from the surrounding highlands to the extensive agricultural farm lands areas were reported as a factor contributing to this epidemic [18]. However, the number of cases reported from 2010 to 2013 has dropped and could be attributed to a lack of awareness for the need of early diagnosis and treatment of VL cases in the health institution.

There are some reports in other countries indicating that early diagnosis and treatment can help in controlling VL outbreaks and transmission [20–22].

There was also a dramatic increase in the number of VL cases detected in 2014, while the number of deaths also decreased from 2013 to 2014.

This may be due to early diagnosis and treatment of VL epidemic cases, as well as increased utilization of the health center when VL clinical signs are seen. Additionally, the number of VL relapse cases dramatically decreased from 2010 onwards, a potential consequence of increased treatment adherence.

Nowadays, the study area is considered to be endemic for VL due to various environmental factors, such as population migration to and from endemic areas, HIV-VL co-infection, and malnutrition.

Hence, complete eradication is a big challenge unless early diagnosis and treatment of cases and integrated prevention and control mechanism are applied in the study area.

## Conclusion

The distribution of VL is still high in the study area. The number of VL relapse cases was dramatically decreased from 2010 onwards; however, there is an increase in the number of VL cases detected in 2014.

Therefore, early case detection using highly sensitive and specific diagnostic techniques, timely analysis, treatment with appropriate therapeutics, and enhanced awareness of preventions and control strategies should be prioritized in endemic areas.

## Data Availability

Data is available upon request.

## Abbreviations

HIV-VL: Human Immunodeficiency Virus and Visceral Leishmaniasis co-infection
NGO: Non-Governmental Organization
PKA: Primary Kala-azar
PKDL: Post Kala-azar Dermal Leishmaniasis
rK_39_: 39-amino-acid-repeat recombinant leishmanial antigen from *Leishmania chagasi*
SOP: Standard operating procedure
VL: Visceral Leishmaniasis
